# New Therapeutic Challenges in Pediatric Gastroenterology: A Narrative Review

**DOI:** 10.3390/healthcare13080923

**Published:** 2025-04-17

**Authors:** Valeria Dipasquale, Claudio Romano

**Affiliations:** Pediatric Gastroenterology and Cystic Fibrosis Unit, Department of Human Pathology in Adulthood and Childhood “G. Barresi”, University Hospital “G. Martino”, 98122 Messina, Italy; romanoc@unime.it

**Keywords:** biologics, celiac disease, enzyme therapy, eosinophilic esophagitis, immunotherapy, inflammatory bowel disease, microbiome, autoimmune hepatitis, personalized medicine, proteomics, small molecules

## Abstract

Pediatric gastroenterology is entering a pivotal phase marked by significant challenges and emerging opportunities in treating conditions like celiac disease (CeD), eosinophilic esophagitis (EoE), inflammatory bowel disease (IBD), and autoimmune hepatitis (AIH) pose significant clinical hurdles, but new therapeutic avenues are emerging. Advances in precision medicine, particularly proteomics, are reshaping care by tailoring treatments to individual patient characteristics. For CeD, therapies like gluten-degrading enzymes (latiglutenase, Kuma030) and zonulin inhibitors (larazotide acetate) show promise, though clinical outcomes are inconsistent. Immunotherapy and microbiota modulation, including probiotics and fecal microbiota transplantation (FMT), are also under exploration, with potential benefits in symptom management. Transglutaminase 2 inhibitors like ZED-1227 could help prevent gluten-induced damage. Monoclonal antibodies targeting immune pathways, such as AMG 714 and larazotide acetate, require further validation in pediatric populations. In EoE, biologics like dupilumab, cendakimab, dectrekumab (IL-13 inhibitors), and mepolizumab, reslizumab, and benralizumab (IL-5/IL-5R inhibitors) show varying efficacy, while thymic stromal lymphopoietin (TSLP) inhibitors like tezepelumab are also being investigated. These therapies require more pediatric-specific research to optimize their use. For IBD, biologics like vedolizumab, ustekinumab, and risankizumab, as well as small molecules like tofacitinib, etrasimod, and upadacitinib, are emerging treatments. New medications for individuals with refractory or steroid-dependent AIH have been explored. Personalized therapy, integrating precision medicine, therapeutic drug monitoring, and lifestyle changes, is increasingly guiding pediatric IBD management. This narrative review explores recent breakthroughs in treating CeD, EoE, IBD, and AIH, with a focus on pediatric studies when available, and discusses the growing role of proteomics in advancing personalized gastroenterological care.

## 1. Introduction

Pediatric gastroenterology is on the cusp of an exciting era marked by both unprecedented challenges and remarkable opportunities. As the understanding of gastrointestinal diseases deepens, so does the understanding of the complexity of their diagnosis and treatment.

Celiac disease (CeD), eosinophilic esophagitis (EoE), inflammatory bowel disease (IBD), and autoimmune hepatitis (AIH), together represent only a fraction of the diverse spectrum of gastrointestinal disorders affecting children worldwide. Although these diseases differ significantly in their etiology and clinical manifestations, they have one thing in common: they represent significant clinical challenges for pediatric gastroenterologists. Despite significant advances in recent years, many patients continue to experience suboptimal outcomes or face significant treatment-related complications. However, the emergence of novel therapeutic modalities is giving patients and physicians new hope. From targeted biologics to innovative nutritional interventions, these new treatments promise to transform the landscape of pediatric gastroenterology. The emergence of precision medicine has ushered in a new era of personalized care, allowing physicians to tailor treatment strategies to the unique genetic, molecular, and clinical characteristics of individual patients. At the heart of this paradigm shift is the emerging field of proteomics, which aims to elucidate the complex interplay between proteins and disease pathogenesis. By harnessing the power of proteomic profiling, pediatric gastroenterologists are able to gain invaluable insights into disease mechanisms, biomarker discovery, and therapeutic targeting, paving the way for more effective, patient-centered interventions.

The aim of this article was to provide a comprehensive examination of these transformative developments, focusing on recent advances in the treatment of CeD, EoE, and IBD. Pediatric data were included when available. Additionally, we examine the evolving role of proteomics in pediatric gastroenterology and its implications for precision medicine. The most relevant papers were identified through a comprehensive PubMed/Medline search, with no time restrictions and giving priority to the most recent publications. Additional papers were identified by reviewing the reference lists of retrieved publications.

## 2. Methods

Relevant studies published over the last 10 years were identified via a PubMed/Medline (http://www.ncbi.nlm.nih.gov/pubmed/ (accessed on 1 December 2024) search using the keywords or combinations of keywords: celiac disease, eosinophilic esophagitis, inflammatory bowel disease, autoimmune hepatitis, enzyme therapy, immunotherapy, zonulin inhibitors, nanotechnology, monoclonal antibodies, transglutaminase 2 inhibitor, biologics, small molecules, proteomics, microbiome, and personalized medicine. Clinical trials were retrieved from the main clinical trials databases (www.clinicaltrials.gov and www.clinicaltrialsregister.eu) (accessed on 1 December 2024). Particular emphasis was placed on clinical trials, cohort, case–control, and cross-sectional studies. Pediatric data were included when available. Additional studies were identified by reviewing citations and references of relevant publications. Non-English publications were excluded. A systematic approach to study selection was not implemented. Instead, data were extracted based on their relevance to the topic.

## 3. Celiac Disease

The CeD is a chronic immune-related disorder triggered by gluten intake. Genetically susceptible people who consume gluten are affected and the disease may develop in individuals of any age. The global prevalence of CeD is 1.4% and 0.7% with data coming from serological testing and biopsy data, respectively [1]. From a recent meta-analysis and systematic review, it has emerged that CeD is becoming more prevalent in children throughout Europe, making it one of the most common immune-mediated gastrointestinal diseases [2]. The clinical presentation of CeD is variable including symptoms from typical diarrhea to asymptomatic cases, with intestinal permeability, poor nutrition, and hyposplenism increasing the risk of hospitalizations and infections [3]. For certain patients, the European Society of Pediatric Gastroenterology, Hepatology and Nutrition (ESPGHAN) advises diagnosis without biopsy [4]. A strict diet avoiding all gluten-containing foods, including barley, wheat and rye, is considered first-line treatment. The ESPGHAN Gastroenterology Committee (Special Interest Group for Celiac Disease) published recommendations in April 2024 to help children follow a gluten-free diet (GFD) safely [5]. Following a GFD correctly may be difficult for some, as well as being expensive. Moreover, lack of compliance could result in nutritional deficiencies. Thus, non-dietary treatment for CeD should be investigated. Progress in the understanding of the immunopathogenesis of CeD has seen advances in several therapy options that could be adopted as adjuncts or alternatives to GFD [6]. Certain therapies target decreasing the immunogenicity of grains containing gluten, altering the grain or utilizing oral enzymes to degrade immunogenic peptides that are not broken-down during digestion. Moreover, the absorption of peptides can be hindered, their binding to CeD-specific antigen-presenting molecules regulated, and an increase in the immunogenicity of gluten peptides avoided by preventing tissue transglutaminase.

### 3.1. Enzyme Therapies

A further treatment for CeD currently being studied is enzyme treatments [7]. The ultimate aim is to decrease the immunogenicity of gluten-containing grains by breaking down immunogenic peptides that ordinarily are not broken down after digestion into safe fragments that can be tolerated. Prolylendopeptidases (PEP), synthetic glutenases (Kuma030), barley seed-derived glutamine-specific cysteine endoprotease (EP-B2 or ALV001), and (food-grade) subtilisins (Sub-A) are among the fungal, bacterial, and plant glutenases that hold out much promise as a further oral enzyme therapy [7]. The therapeutic application of enzymes may be difficult due to autodegradation and instability. Nevertheless, diverse paths are being studied, including the combination of enzymes with varying optimal pH values with the aim of increasing activity and digestion in both neutral and low pH conditions [7]. To date, latiglutenase (ALV003) appears to yield the best clinical results with adjunctive therapy potential for CeD. It is a 1:1 combination of EP-B2 (ALV001) and SC-PEP (ALV002) [7,8]. ALV003 has shown a substantial protective effect against the development of intraepithelial lymphocytosis and villous atrophy in CeD patients in remission. These patients participated in phase 1b-2a clinical studies, (the CeliAction study included) and were tested with the enzymes + gluten, as opposed to placebo + gluten [9,10]. Conversely, in another study to evaluate efficacy and safety of ALV003 in 494 patients with CeD in North America and Europe no benefits were observed. The study was a double-blind, placebo-controlled, dose-ranging study, and patients were on a strict GFD still having villous atrophy and abdominal discomfort. The participants were randomized to receive the combined enzymes for 12 or 24 weeks at different doses as opposed to placebo. The results of the study reported only a slight improvement in both the placebo and enzyme treated groups [11]. From the study it emerges that patients who follow a stringent GFD in a clinical trial environment may increase diet adherence because of the trial setting itself and, thus, also small intestine histology improves [11]. Moreover, it was seen that ALV003 is ineffective in improving intestinal histology. However, notwithstanding the disappointing outcome, the enzyme combination cannot be ruled out as an adjuvant treatment to other pharmacological approaches: ALV003, taken with meals, helped a subgroup of patients with positive transglutaminase (TG)-2 autoantibodies to enhance both quality of life (QoL) and symptoms, as was seen from a sub-analysis of the CeliAction study [12].

Molecular modeling and chemical modification of enzymes through encapsulation and/or attachment of groups such as polyethylene glycol (PEG) are further methods to support the therapeutic use of enzymes [7]. The first technique attempts to create a new enzyme that is active under acidic conditions while introducing the desired substrate selectivity through genetic modification. The modified enzyme, known as Kuma030, outperformed ALV003 in vitro [13]. Kuma030 is currently being evaluated in a phase 1–2 clinical trial (NCT03409796) as a new enzyme therapy for CeD. The spectrum of gluten-degrading enzymes is expanding and their potential therapeutic use for the treatment of CeD is encouraging. Further research is needed to determine the medium- and long-term safety and effectiveness of enzyme therapy. To date, no studies have been designed for pediatric CeD patients.

### 3.2. Immunotherapy

Immunotherapy for CeD requires the development of gluten tolerance. The aim is to reduce or eliminate gluten-induced immunological reactions, thereby alleviating symptoms and preventing intestinal damage. Several research initiatives and clinical trials are investigating immunotherapy options for CeD, including oral immunotherapy (OIT), sublingual immunotherapy (SLIT), and epitope-specific immunotherapy [6]. OIT is the gradual introduction of modest, increasing amounts of gluten into the diet with the aim of desensitizing the immune system and inducing gluten tolerance. With SLIT, small amounts of gluten are placed under the tongue and absorbed through the oral mucosa. OIT and SLIT procedures often begin with low doses of gluten that are gradually increased over time under medical supervision until a maintenance dose is reached. SLIT may have advantages over OIT, including a lower risk of gastrointestinal side effects and a simpler method of administration. The STANFORD study (NCT04037110) is a phase 2 clinical trial evaluating the safety and efficacy of experimental oral immunotherapy to establish immunologic tolerance to gluten in adults with CeD. The aim of the study is to evaluate the effects of OIT on gluten tolerance, immune response, and symptoms in participants with CeD.

Epitope-specific immunotherapy modifies or selectively blocks specific gluten peptides (i.e., epitopes) identified by the immune system in CeD patients [6,14]. Several options are being explored, including peptide-based vaccinations, monoclonal antibodies that target specific epitopes, and genetic engineering strategies to modify gluten proteins. The TIMP-GLIA study (NCT04530123) is a phase 2 clinical trial evaluating the safety and efficacy of OIT with TIMP-GLIA, a combination therapy consisting of two recombinant gluten-specific T cell epitopes, in patients with CeD. The aim of the study is to evaluate the effects of TIMP-GLIA on gluten tolerance, immunological responses, and intestinal histology in CeD patients.

An epitope-specific vaccine is currently under investigation and contains five different immunodominant gluten peptides that commonly activate the immune response in individuals with CeD. The aim is to establish immunological tolerance to gluten in CeD patients and thus avoid the inflammatory reaction and tissue damage that occurs in CeD. A phase 1 clinical trial of this vaccine showed that it was generally well tolerated, and side effects were mild to moderate [15,16]. Immunological analyzes confirmed that this epitope-specific vaccine induced specific T cell responses to gluten peptides and modulated immune markers associated with CeD [15,16]. Clinical results showed that this vaccine reduced gluten-induced immune responses and symptoms in individuals with CeD, supporting its potential as a therapeutic vaccine against CeD [7,15,16]. However, the clinical trial has been discontinued due to results that showed that the vaccine to treat CeD did not provide protection from gluten exposure when compared to a placebo. Further research is needed to optimize vaccine formulation, dosing regimen, and treatment protocols, as well as to assess long-term safety and effectiveness in larger patient groups.

Overall, although immunotherapy holds promise as a treatment for CeD, further research is needed to optimize protocols, increase efficacy, and ensure long-term safety and tolerability. Patients seeking CeD immunotherapy should meet with their healthcare providers to learn about available alternatives, potential risks and benefits, and tailored treatment strategies. To date, no studies have been designed for pediatric CeD patients.

### 3.3. Zonulin Inhibitors

Researchers are studying zonulin inhibitors as a possible therapy to improve intestinal barrier function and reduce gluten sensitivity. Zonulin is a protein that controls the permeability of tight junctions between epithelial cells in the intestine. In people with CeD, zonulin levels are elevated, leading to leaky gut and gluten intolerance. Zonulin inhibitors restore the integrity of the intestinal barrier by inhibiting zonulin activity, thereby reducing the passage of gluten and other potentially harmful compounds through the intestinal barrier [17]. Several zonulin inhibitors have been evaluated in preclinical and early-phase clinical trials for the treatment of various gastrointestinal diseases, including CeD [18].

Larazotide acetate (AT-1001) is one of the most extensively studied zonulin inhibitors. It is a synthetic version of a naturally occurring zonulin inhibitor that has been tested in clinical trials for its ability to reduce intestinal permeability and relieve symptoms in CeD patients [18]. A phase 2 clinical trial evaluated the safety and efficacy of larazotide acetate in combination with a GFD in 184 adult CeD patients [19]. Participants were randomly assigned to receive larazotide acetate or placebo for six weeks in addition to a GFD. The main outcome measure was change in intestinal permeability, assessed by the urinary lactulose/mannitol ratio. Secondary endpoints included symptoms, quality of life (QoL) and histological markers.

Larazotide acetate could be taken into consideration as an additional therapy for CeD because it was seen to decrease intestinal permeability and improve symptoms in CeD patients when compared to patients receiving placebo [19]. This was confirmed in another study, a phase 2b clinical trial, which looked at the effectiveness and safety of larazotide acetate administered along with a GFD in people with CeD who continued with symptoms [20]. The study population was randomized to either a larazotide acetate or placebo group for 12 weeks, while following a GFD. The aim of the use of larazotide acetate was a decrease in the average number of days per week with moderate to severe symptoms. The study discovered that individuals receiving larazotide acetate suffered fewer symptoms and enjoyed a better QoL than those who received a placebo, indicating possible advantages for symptom management in refractory CeD [20]. A further multicenter, randomized, double-blind, placebo-controlled study assessed the safety and effectiveness of larazotide acetate along with a GFD in 342 adult CeD patients who, notwithstanding a GFD, continued with symptoms [21]. The aim was a change in the average daily Gastrointestinal Symptom Rating Scale (GSRS) score, and larazotide acetate 0.5 mg administration decreased signs and symptoms more effectively when combined with a GFD than alone [21].

These studies provide important information on the potential efficacy and safety of larazotide acetate as adjunctive therapy in CeD [17,18]. However, it is important to highlight that larazotide acetate has not yet been approved for clinical use in CeD and further research is needed to confirm its benefits, define effective dosing regimens, and assess long-term consequences. Other zonulin inhibitors and intestinal permeability modulators are also being investigated in preclinical and clinical studies for their possible involvement in CeD treatment. Further research is needed to assess the therapeutic potential of zonulin inhibitors and to identify which patient subgroups may benefit most from this approach.

### 3.4. Microbiota Modulation

The gut microbiota is critical to immune function and gut health. New research suggests that dysbiosis, or an imbalance in gut flora, may contribute to the development or progression of CeD. Probiotics, prebiotics, and fecal microbiota transplantation (FMT) are being studied as possible strategies for modulating gut microbiota and alleviating symptoms in CeD patients [6,22]. A systematic review and meta-analysis published in 2023 examined the effects of probiotics on CeD, including 14 studies, and concluded that probiotics could alleviate gastrointestinal symptoms and immune response while also improving dysbiosis (increased Bifidobacterium and Lactobacillus) in CeD patients [23]. However, the authors emphasize that further well-designed clinical trials are required to validate these findings and define the most effective probiotic strains, doses, and treatment durations. A recent double-blind, placebo-controlled study of 31 CeD patients randomly allocated to probiotics or placebo found that 12 weeks of probiotic treatment with different strains can modify gut microbiota composition and alleviate gastrointestinal symptoms in CeD patients [24].

Prebiotics have been shown to influence gut microbiota composition and improve intestinal barrier function in various gastrointestinal diseases. There have been few clinical studies specifically examining the effects of prebiotics on people with CeD. A small pilot study examined the influence of a prebiotic supplement (fructo-oligosaccharides) on intestinal permeability and symptoms in 34 children with CeD receiving a GFD [25]. The study found that the use of prebiotics reduced intestinal permeability and improved gastrointestinal symptoms. However, larger clinical trials are needed to confirm these results and evaluate the long-term effectiveness of prebiotic therapy in CeD treatment.

Synbiotics, a mix of both probiotics and prebiotics, have been proposed as a potential method for stimulating gut microbiota and improving gastrointestinal well-being in CeD, but clinical research on synbiotics in CeD is scanty. Eighty-two pediatric patients with suspected CeD participated in a 2020 study which looked at the impact of synbiotics on blood levels of anti-tTG antibodies [26]. Patients were allocated randomly into a synbiotic group who were given a daily dosage, for 20 days, of a synbiotic with probiotics from several strains, and a control group who were not given any medication. Anti-tTG antibody levels were reduced by 73% and 56% in the synbiotic and control groups, respectively [26]. More high-quality evidence is needed to validate these findings and better understand the processes that promote synbiotic effects in CeD.

While FMT has been shown to be effective in the treatment of recurrent Clostridioides difficile infections and other gastrointestinal problems [27], its role in refractory CeD treatment is largely unknown. A case report published in 2018 described a 68-year-old male patient with refractory CeD type II who underwent FMT and demonstrated clinical remission and intestinal histological normalization [28]. However, it is impossible to draw conclusions based on a single case report. Well-designed clinical studies are required to determine the efficacy, safety, and best treatment regimens of probiotics, prebiotics, synbiotics, and FMT in adult and pediatric CeD patients.

#### Considerations on Limitations, Barriers to Clinical Implementation, and Potential Risks of Microbiota Modulation

Because microbiota vary between individuals, a one-size-fits-all strategy may be unsuccessful. Diet, genetics, geography, and other variables can all have a substantial impact on the microbiota composition. Depending on the patient’s unique gut flora, various probiotic strains may function well for one but not for another. This necessitates more tailored treatment plans, which are difficult to standardize in clinical studies. Clinical data supporting such treatments is sparse and contradictory, with some research demonstrating benefits from probiotics and prebiotics but others unable to duplicate similar findings. FMT has shown promise but is still in its early phases, necessitating bigger trials to determine efficacy and safety. Furthermore, approval from regulators for microbiota-based therapeutics is challenging since FMT and microbial manipulation are novel concepts. Regulatory organizations must guarantee that these therapies are safe and do not have negative consequences, such as introducing hazardous bacteria or modifying the immune system in unpredictable ways. Finally, the lack of agreement on which microbial strains or combinations are most beneficial delays the approval procedure.

### 3.5. Nanotechnology

Nanotechnology-based techniques are being investigated to develop targeted drug delivery systems for the treatment of CeD. These systems aim to deliver therapeutic chemicals such as enzymes or immunomodulators directly to the site in the intestine where gluten is released, thereby reducing systemic side effects while increasing effectiveness. Nanotechnology research in CeD is a growing field with numerous potential applications. Nanotechnologies open new perspectives for targeted drug delivery, improved diagnostics, and better knowledge of disease mechanisms in CeD. Potential applications include (i) nanoparticle-based drug delivery, which could deliver therapeutic agents such as gluten-degrading enzymes or immunomodulatory drugs directly into the intestine, thereby minimizing systemic side effects and enhancing therapeutic effects; (ii) nanoparticle-based gluten sensors and assays that can be designed to detect gluten peptides or antibodies to gluten with high sensitivity and specificity, enabling rapid and reliable gluten detection in food samples or biological fluids; (iii) nanoparticle-based imaging techniques offer the potential for non-invasive visualization of intestinal damage in individuals with CeD; (iv) nanoparticle-based vaccine delivery technologies that can improve the immunogenicity and efficacy of vaccines by promoting antigen uptake and presentation to immune cells [29,30]. Research is currently underway to develop nanoparticle-based vaccine delivery systems for CeD immunotherapy, including liposomes, polymeric nanoparticles, and virus-like particles.

#### Considerations on Limitations, Barriers to Clinical Implementation, and Potential Risks of Nanotechnology

Although there is great potential for using nanotechnology to treat celiac disease and other disorders, there are considerable obstacles to its use, including concerns about toxicity, regulatory issues, and the viability of large-scale production. The small size of nanoparticles may cause toxicity difficulties since they may accumulate in organs like the kidneys, liver, or lungs. Inflammation or immunological activation may result from their interactions with the immune system. There is a lack of safety data and an incomplete understanding of long-term consequences, which raises concerns about their use in medicine. The regulatory framework for nanotechnology in medicine is still developing. Regulatory agencies have yet to establish clear guidelines for nanoparticles, creating challenges in safety assessment, quality control, and approval. Enhancing the production of nanoparticles from clinical trials to large-scale manufacturing is also a challenging task. The heterogeneity in size, shape, and surface qualities might impact safety and therapeutic effects, making it difficult to ensure consistent quality and repeatability. The cost of production is also significant since it requires specialized equipment and materials.

### 3.6. Monoclonal Antibodies

Monoclonal antibodies that target specific components of the immune response to gluten are being investigated as potential therapies for CeD [6]. These antibodies can reduce inflammation and protect the intestinal lining from gluten-related damage. Monoclonal antibodies provide a viable treatment option for CeD by targeting specific immunological mechanisms involved in disease etiology. A possible target molecule is IL-15, a proinflammatory cytokine involved in CeD development [31]. Monoclonal antibodies against IL-15 or its receptor have been postulated as a potential therapeutic method to inhibit IL-15-mediated immune activation and tissue damage in the intestine. A phase 2 clinical trial evaluated the safety and efficacy of AMG 714, a monoclonal antibody targeting IL-15, in 64 adult CeD patients following a GFD [32]. The aim of the study was to evaluate the influence of AMG 714 on CeD-related intestinal histology (i.e., villus height to crypt depth ratio, CD3-positive intraepithelial lymphocyte density), symptoms, and immunological markers. The results of this study were mixed and suggested that further research on AMG 714 in patients with unresponsive CeD is warranted. Monoclonal antibodies are expensive to produce and using them to treat CeD would likely result in significant costs for both individuals and healthcare systems. Until long-term clinical data supports their safety and efficacy, their cost-effectiveness remains uncertain. Although monoclonal antibodies have demonstrated potential in the treatment of CeD, their long-term safety is yet unknown. Concerns have been raised regarding the long-term consequences of monoclonal antibodies’ interactions with the immune system, such as immune system dysfunction, negative reactions, or unexpected side effects. Before they may be generally advised for CeD patients, extensive, long-term clinical trials are required to completely evaluate their safety and effectiveness.

### 3.7. Transglutaminase 2 Inhibitor

Small intestine transglutaminase 2 in CeD deamidates glutamine residues in gluten peptides, promoting T cell activation and inducing mucosal damage. A selective oral transglutaminase 2 inhibitor called ZED-1227 is being developed to treat CeD and non-alcoholic fatty liver disease (NAFLD). The drug candidate is administered orally in the form of a capsule and acts by targeting transglutaminase (peptidomimetic agent). In subjects with well-controlled CeD who undertook a daily gluten challenge, a 6-week therapy with ZED1227 at three dosage levels reduced gluten-induced duodenal mucosal damage at all three dose levels when compared with placebo in a proof-of-concept trial [33]. The incidence of adverse events (vomiting, headache, nausea, diarrhea, and stomach discomfort) was comparable in every group [33]. Further research with a larger patient population is required to confirm the safety and efficacy of ZED1227.

## 4. Eosinophilic Esophagitis

EoE is a chronic inflammatory disease of the esophagus, characterized by esophageal dysfunction and eosinophilic inflammation. A recent comprehensive review and meta-analysis found that the global pooled incidence and prevalence of EoE were 5.31 and 40.04 cases per 100,000 population years, respectively [34]. It is worth noting that the incidence of EoE has gradually grown from 1976 to 2022 [34]. EoE therapy offers a number of obstacles due to its complexity and the absence of an internationally accepted standard of care. One of the most significant issues is the lack of a clear treatment for EoE, which necessitates long-term care methods to control symptoms and prevent complications. Dietary modification is the foundation of EoE therapy, with the primary goal of removing trigger foods. However, identifying and avoiding particular allergens can be challenging and time-consuming [35]. Furthermore, adhering to a rigid exclusion diet may be difficult or ineffective for many individuals, especially if several food triggers exist. Pharmacological therapies, such as proton pump inhibitors (PPIs) or topical corticosteroids, are frequently used to reduce inflammation and symptoms in EoE [35].

Several novel drug treatments targeting specific pathways involved in EoE development are being investigated, including anti-interleukin (IL)-4, IL-13, and IL-5 monoclonal antibodies and thymic stromal lymphopoietin (TSLP) inhibitor signaling pathways [36]. These drugs are intended to modify immunological responses and reduce eosinophilic inflammation in the esophagus. Notably, research on other eosinophilic disorders is scarce, with most studies focusing on broader eosinophilic gastrointestinal diseases like EoE. Despite their focus on EoE, existing trials may provide a comparable basis for evaluating the use of novel medical products in other eosinophilic gastrointestinal diseases [37]. Translational research has discovered promising targets for the treatment of eosinophilic gastritis and enteritis, such as mast cells and eosinophils. So far, there are no agents that have been evaluated for eosinophilic colitis [37].

### 4.1. Dupilumab

Dupilumab is an IgG4 monoclonal antibody binding to the IL-4 receptor alpha subunit [38]. It was initially authorized in May 2022 for the treatment of several allergic disorders such as atopic dermatitis and asthma in adults and children aged 12 and over [39]. The FDA then (January 2024) expanded the indication for it for the treatment of EoE in pediatric patients aged 1 to 11 years and weighing at least 15 kg making this clearance the first therapy for EoE in this age group [40]. The clearance was grounded on results from the phase 3, randomized, double-blind, placebo-controlled EoE-KIDS research (two phases), which assessed the safety and effectiveness of dupilumab in children under 12 [30,36]. At least one type 2 inflammatory condition was present, initially, in 97% of participants. Part A (study first phase) of the study saw 61 participants receive double-blind treatment for 16 weeks. In part B (second phase), 47 children from Part A were included in a 36-week extension of active therapy. Children who were administered dupilumab in Part A stayed on it, while children on placebo were switched to dupilumab. At the conclusion of Part A, 66% of the 32 children who received a higher dose of dupilumab, which depended on their weight, had achieved histologic disease remission, the prime aim, as against just 3% of the children on placebo [41]. At week 52, just over half (53%) of the children treated in Parts A and B who had achieved histologic remission remained so. Furthermore, at week 52, 53% of children in the placebo group in Part A who were switched to dupilumab in Part B were still in histologic remission. Based on responses to the Pediatric EoE Signs/Symptoms Questionnaire-caregiver version, there was a higher decrease in the percentage of days with one or more indications of EoE at week 16 in children who received dupilumab than in children who received the placebo [41,42]. In patients over the age of 12, the 24-week safety profile was comparable to that of Part A. The most frequent adverse events linked to dupilumab were upper respiratory tract infections, injection site reactions, herpes virus infections, and arthralgias.

### 4.2. Cendakimab and Dectrekumab

Cendakimab (RPC4046, CC-93538), a humanized IgG1k monoclonal antibody against IL-13, displayed potential efficacy in a multicenter trial (HEROES) of adult patients with active EoE. Weekly subcutaneous injections of 180 mg or 360 mg substantially improved histopathological and endoscopic results at week 16 as compared to placebo [43]. Although dysphagia did not improve considerably, the high dosage group exhibited benefits on the overall rating of disease severity. Common side effects were headaches and upper respiratory tract infections. These results were validated by an open-label extension, which also suggested a possible role in reducing the fibrostenotic risk in EoE [44]. Ongoing studies will provide further insight, including adolescent studies and drug interaction studies [35]. Dectrekumab (QAX576), another anti-IL-13 monoclonal antibody, was tested in an EoE proof-of-concept trial [45]. Adult patients received three monthly intravenous injections of dectrekumab (6 mg/kg). Although there was a significant decrease in esophageal eosinophil counts compared to placebo, the primary objective of response rate was not met. However, improvements in the expression of key mediators involved in EoE-induced inflammation were noted [45]. Dectrekumab is also being investigated for other Th2 diseases, including Crohn’s disease and keloids [36].

### 4.3. Mepolizumab, Reslizumab, and Benralizumab

Due to their critical involvement in eosinophilic inflammation, IL-5 and its receptor (IL-5-R) have long been studied in the treatment of EoE. Th2 cells, mast cells, and eosinophils largely release IL-5, which controls eosinophil activities and contributes to epithelial remodeling and esophageal motility disorder. Studies demonstrate a complex relationship between IL-5 levels and disease severity, suggesting multiple phenotypes of EoE development [46]. Mepolizumab and reslizumab, humanized IgG1k monoclonal antibodies targeting circulating IL-5, showed histopathological improvements in EoE but had limited clinical efficacy in both adults and children [47,48,49]. Benralizumab, targeting IL-5-R, similarly produces antibody-dependent cell-mediated cytotoxicity with promising results but no direct association between histopathological and clinical benefits in trials (the MESSINA study; NCT04543409). The nonlinear relationship between histopathological and clinical improvements questions the role of eosinophilic inflammation in EoE and prompts additional investigation of other Th2-like mediators in mucosal inflammation [36].

### 4.4. Drugs Acting on the Thymic Stromal Lymphopoietin

TSLP, an IL-2 cytokine, functions as an alarmin and regulates the Th2 response by promoting the production of numerous cytokines and producing proinflammatory effects. Epithelial cells are the main producers. TSLP interacts with a receptor composed of IL-7R and TSLP-R chains and affects different immune cell types. Recent studies have found greater TSLP receptor sensitivity in esophageal-derived memory CD4+ T cells from EoE patients, suggesting a role in the pathophysiology of the disease [50]. While specific investigations with TSLP inhibitors in EoE are limited, these findings highlight the need of future research into the function of TSLP in EoE pathogenesis, as well as the potential utility of controlling the TSLP pathway as a novel treatment approach for the condition. Tezepelumab is a human IgG2 monoclonal antibody that binds to circulating TSLP and blocks its interaction with the receptor. The US FDA has designated tezepelumab as an orphan medication for the treatment of EoE [51], and current trials seek to assess its effectiveness and safety in adults and adolescents with EoE.

## 5. Inflammatory Bowel Disease

IBD includes Crohn’s disease (CD), ulcerative colitis (UC), and unclassified IBD, which are chronic inflammatory diseases of the gastrointestinal tract with a relapsing-remitting course and a wide spectrum of clinical symptoms. In 2016, IBD affected 1 in 209 people and 1 in 1299 children aged 2 to 17 years in the United States [52]. The 2019 Global Burden of Disease Study identified 4.9 million cases of IBD in 204 countries, with China and the US leading the way [53]. Although the exact cause of IBD is unknown, the pathogenesis is thought to be related to the host’s genetic predisposition, intestinal flora, various lifestyle factors (such as diet, smoking, and physiological stress), and immunological imbalances [54,55,56]. While biologic drugs have transformed the treatment of IBD in adults and children, more research and innovation are needed to address unmet needs such as refractory disease, mucosal healing, long-term complication prevention, and remission maintenance. New therapies, including novel biologics, small molecules, and microbiome-based interventions, hold promise for improving outcomes in IBD, but further research is needed to evaluate their safety and effectiveness, particularly in pediatric populations.

### 5.1. New Biologics

#### 5.1.1. Vedolizumab

Vedolizumab is a monoclonal antibody that is used to treat IBD in adult patients who have not responded to conventional therapy or have had adverse effects from prior medications [57,58]. Although vedolizumab is not currently licensed for pediatric IBD in all areas, it has been researched in this group. The VEDOIBD trial, phase 3 research, looked at the use of vedolizumab in pediatric patients aged 6 to 17 years with moderate to severe CD [59].

The study found high clinical remission rates and improvements in multiple disease activity markers when compared to placebo. Other trials have found that vedolizumab had positive effects in pediatric CD patients, including steroid-free remission and mucosal healing [60]. Vedolizumab has also been studied in clinical trials for pediatric UC [60]. It has shown a generally acceptable safety profile in pediatric IBD patients, with side effects comparable to those seen in adults. Common side effects include headache, nasopharyngitis, stomach discomfort, and upper respiratory tract problems [58,61]. A 2024 retrospective assessment of pediatric patients with IBD treated with vedolizumab between 2018 and 2022 showed that proactive therapeutic drug monitoring (TDM) and early dosage adjustment with vedolizumab enhanced drug durability and clinical outcomes in pediatric patients with IBD [62].

Vedolizumab represents a valuable treatment option for children and adolescents with moderate to severe disease who have not responded to conventional therapies and can be used off-label in clinical practice based on available evidence and physician discretion. Regulatory authorities may require additional data or specific pediatric studies before granting formal approval for pediatric indications.

#### 5.1.2. Ustekinumab

Ustekinumab, a monoclonal antibody that suppresses interleukin-12 (IL-12) and IL-23, is a relatively newly introduced biologic, and has been licensed as therapy in adults for moderate to severe CD and UC (63). While not commonly accepted as therapy in juvenile IBD in all areas, studies have been undertaken to assess its efficacy and safety in this pediatric population. From a 2023 comprehensive review, low-quality data, above all from case series and cohort studies, revealed that around half of pediatric patients with CD and UC attained remission within a year with an acceptable safety profile [63]. An IM-UNITI study examined a subset of patients (12 to 17 years) with moderate to severe CD who failed to respond to conventional therapy or TNF-alpha inhibitors. The study demonstrated high clinical remission rates and improvements in a variety of disease activity markers, when compared to the placebo group [64]. On the contrary, there is scant evidence supporting the use of ustekinumab in pediatric UC. In a recent retrospective chart analysis, 10 children with UC who received ustekinumab were investigated [65]: 9 experienced steroid-free clinical and biochemical remission; 7 had a follow-up colonoscopy, and 6 obtained endoscopic remission [65]. Information on long-term benefits and high-quality proof are still required.

#### 5.1.3. Risankizumab

Risankizumab, a monoclonal antibody specifically targeting the p19 component of IL-23, is a cytokine that is implicated in the pathogenesis of IBD [66]. Clinical trials have been conducted to assess the efficacy and safety of risankizumab in an IBD population, despite it not yet being licensed for IBD therapy. Studies have demonstrated that intravenous risankizumab was effective and well tolerated as an induction therapy in patients (16 to 80 years) with moderately to severely active CD who were intolerant or inadequately responded to one or more approved biologics or conventional therapy (ADVANCE Study) or biologics (MOTIVATE Study) [67]. Recently, a published FORTIFY study was a phase 3, multicenter, randomized, double-blind, placebo-controlled maintenance withdrawal study that discovered that subcutaneous risankizumab was safe and efficacious in sustaining remission in a similar group [68]. A phase 3, double-blind, placebo-controlled trial (INSPIRE Project) aimed to assess the effectiveness and safety of risankizumab as an induction treatment in patients with moderately to severely active UC. This monoclonal antibody significantly improved clinical remission, clinical response, and endoscopic remission at week 12 compared to placebo administration [69]. Patients in the INSPIRE study were unable to tolerate or respond adequately to standard and/or advanced UC treatment.

Additional research, including real-world trials, is required to validate these findings and verify the long-term effectiveness and safety of risankizumab in IBD patients.

Table 1 summarizes the key details about vedolizumab, ustekinumab, and risankizumab for the treatment of IBD.

#### 5.1.4. Considerations on Current Evidence Gaps and Challenges

Real-world data gaps for novel biologics like vedolizumab, ustekinumab, and risankizumab limit our understanding of their long-term efficacy and safety, especially in different patient populations with comorbidities. While clinical trials show promising results, real-world variability in patient responses needs more attention. Additionally, TDM, which is essential to optimize dosing and detect antibody development, is hindered by cost and limited availability. The challenges of access and affordability also persist, as these biologics are expensive, and insurance coverage or geographical limitations often restrict access. Addressing these issues through long-term studies, expanded TDM, and cost-reduction strategies like biosimilars is crucial for improving patient outcomes and ensuring wider accessibility.

### 5.2. Small Molecules

Initial research would seem to suggest that small molecules may be a significant and developing treatment option for pediatric IBD and might be useful additions or alternatives to traditional biological therapy in pediatric CD and UC [70]. Tofacitinib is a little studied oral janus kinase (JAK) inhibitor in children and adolescents with moderate to severe UC. The safety, efficacy, and pharmacokinetics of the JAK inhibitor is presently under exam in an on-going open-label phase 3 study (NCT04624230) in pediatric patients (2 to 17 years) with moderately to severely active UC, defined by a Mayo score of at least 6, a rectal bleeding score of at least 1, and an endoscopic subscore of at least 2. Etrasimod is an oral, single daily-dose, selective sphingosine-1-phosphate1,4,5 receptor modulator used to treat moderately to severely active UC [71]. In the ELEVATE UC 52 and ELEVATE UC 12 studies, two independent randomized, multicenter, double-blind, placebo-controlled phase 3 trials, etrasimod was found to be safe and effective as an induction and maintenance treatment in adults with moderately to severely active UC who failed to respond well or could not tolerate at least one authorized UC treatment [71]. A recent post hoc analysis from these phase 3 trials showed that UC patients, including those with isolated proctitis (<10 cm rectal involvement), enjoyed substantial improvement at weeks 12 and 52 having received etrasimod, compared to the placebo group. Patients had been administered etrasimod or placebo and randomized at 2:1 [72]. Upadacitinib is a novel selective JAK 1 inhibitor that has shown efficacy in the management of moderate to severe IBD and has received Food and Drug Administration approval for UC. Upadacitinib showed to be rapidly effective and safe in medically resistant patients with UC or CD, including in those who had prior tofacitinib exposure [73].

## 6. Autoimmune Hepatitis

AIH is a chronic liver disease caused by immune-mediated inflammation of the liver. Although AIH is relatively uncommon in adulthood, it remains one of the primary causes of chronic liver disease and liver transplantation. The estimated prevalence rate is 1.28–15.65/100,000, and the prevalence rate among children is significantly lower than that in adults [74]. Recent developments in the treatment of AIH have resulted in the development of new medications, especially for individuals with refractory or steroid-dependent illness. Rituximab, a monoclonal antibody that targets CD20-positive B cells, has demonstrated encouraging outcomes in both adults and children who are resistant to traditional immunosuppressive treatments such as corticosteroids and azathioprine [75,76]. Clinical trials have shown that rituximab can successfully reduce liver inflammation, normalize liver enzymes, and improve long-term results, particularly in pediatric patients. Another promising drug is mycophenolate mofetil, which has been shown to be an effective alternative to steroids, especially for people who are steroid intolerant [77]. Furthermore, JAK inhibitors, such as tofacitinib, are being investigated in clinical studies, with promising early results in enhancing liver function and lowering disease activity [78].

Several limitations remain in the clinical application of these novel treatments for AIH. One key difficulty is a paucity of long-term evidence on the safety and effectiveness of these treatments, particularly in pediatric populations. While rituximab has shown beneficial in both adults and children, it is not without hazards, such as infections and probable long-term B-cell depletion [75]. Furthermore, mycophenolate mofetil, while beneficial, can have adverse effects such as gastrointestinal problems and bone marrow suppression, restricting its usage in some individuals [77]. JAK inhibitors are still in early phases of clinical studies, with unknown long-term results and the possibility of adverse effects such as thrombosis and infections [78]. Furthermore, the high cost of these biologics prevents wider use, particularly in low-resource settings. Future research should focus on improving AIH treatment algorithms, identifying biomarkers to predict treatment response, and undertaking long-term studies to confirm the safety and efficacy of these innovative medicines in varied patient groups. Furthermore, tailored medicine strategies that consider genetic and immunological characteristics may assist in improving treatment results and reduce side effects in the future.

## 7. Personalized Therapy in Complex Gastrointestinal Diseases

Personalized therapy for complex diseases involves tailoring treatment strategies to individual patients based on their unique clinical, genetic, immunological, and environmental characteristics. Several key aspects contribute to the personalized therapy of these diseases and are summarized below.

### 7.1. Precision Medicine

Precision medicine approaches leverage advances in genetics, molecular biology, and omics technologies to identify biomarkers, genetic variants, and disease mechanisms that underlie individual variability in disease susceptibility, disease progression, and treatment response [79]. Despite the fact that genetics can influence IBD at any age, patients with very early onset IBD (VEO-IBD) are more likely to have monogenic etiologies than those with an older diagnosis. VEO-IBD is defined as clinical symptoms and/or acquiring the diagnosis when younger than 6 years of age. Since 2009, many pediatric patients have been shown to have single gene defects, and some of the findings have resulted in targeted therapy [80]. Exome sequencing, combined with mitochondrial DNA analysis, has revolutionized the diagnosis and treatment of monogenic VEO-IBD. Exome sequencing allows for the identification of pathogenic mutations in genes related to the immune system or other cellular functions. Notable genes linked to VEO-IBD include IL10, IL10RA, IL10RB, and X-linked inhibitor of apoptosis (XIAP). Identifying the underlying genetic mutation allows for personalized treatment strategies tailored to the specific defect. For example, mutations in IL10 and its receptors may require targeted therapies such as IL10 receptor agonists or immunosuppressants. Conversely, a diagnosis of XIAP deficiency may benefit from stem cell transplantation [80,81,82]. In addition to nuclear DNA, mitochondrial DNA (mtDNA) can be involved in some rare cases of VEO-IBD, especially those with mitochondrial dysfunction. mtDNA analysis is critical when mitochondrial dysfunction is suspected, particularly in cases where the clinical presentation includes atypical IBD features such as early onset, multi-organ involvement, or neurodevelopmental issues [83]. Mitochondrial DNA analysis can aid in identifying pathogenic mutations in the mitochondrial genome, providing essential insights into the underlying cause of disorders such as IBD and guiding treatment strategies. Because mtDNA is maternally inherited, this investigation can also trace the inheritance pattern within families, providing critical information regarding the likelihood of recurrence for future generations, particularly in cases where mitochondrial malfunction is passed down through the maternal line [83].

Integrating genetic data such as human leukocyte antigen (HLA) typing, pharmacogenomics, and gene expression profiles with clinical parameters can help stratify patients by disease subtype, severity, and likelihood of response to specific therapies.

Precision medicine relies on advanced technologies such as genomics, bioinformatics, and personalized diagnostics. The integration of these technologies into clinical settings may require infrastructure upgrades, sophisticated data analysis capabilities, and interoperability between systems, which can be challenging to implement in existing healthcare frameworks. Moreover, genetic testing and personalized treatments are costly, and insurance coverage is often limited, raising financial concerns for both patients and healthcare systems. Precision medicine also raises ethical concerns related to data privacy, consent, and potential discrimination based on genetic information. Furthermore, the equitable distribution of precision medicine must be ensured to prevent disparities in access based on socioeconomic status, race, or geography.

### 7.2. Risk Stratification

Risk stratification models aim to predict disease outcomes and treatment responses based on clinical, laboratory and genetic factors [79]. Identifying patients at higher risk of disease progression, complications, or treatment failure allows for targeted interventions, close monitoring, and early escalation of therapy if necessary. For example, risk stratification tools in IBD, such as the Montreal and Paris classifications [84,85] and the PROTECT score [86], help guide treatment decisions and optimize outcomes.

### 7.3. Personalized Treatment Algorithms

Personalized treatment algorithms take individual patient characteristics, preferences, and care goals into account when selecting therapeutic interventions [79]. Treatment decisions can be guided by disease phenotype, severity, location, and behavior, as well as patient preferences, lifestyle factors, and comorbidities. Tailoring therapy to match the specific needs and priorities of each patient improves treatment adherence, satisfaction, and long-term outcomes. Notably, a prognostic biomarker that guides therapy by evaluating outcomes in patients randomized to either top-down or accelerated step-up treatment strategies was found to have no clinical utility in newly diagnosed active CD adults in the PROFILE (PRedicting Outcomes For Crohn’s disease using a moLecular biomarker) study [87]. Another important aspect is the recent trend of combining two biological agents or a biological agent with another medication that falls within the category of so-called “small molecules” (dual biologic therapy) [88]. On the other hand, combination therapy (i.e., the combination of a biological agent with a medication, such as an immunosuppressant) has been used for a number of years. Currently, dual biological therapy and combination therapy are used to treat individuals who are resistant to treatment or who have extraintestinal symptoms that do not improve with traditional therapy. Despite being positive, the current evidence is insufficient given the number of patients who have been included thus far, and further research is required [88].

### 7.4. Therapeutic Drug Monitoring

TDM measures drug levels and anti-drug antibodies to optimize the dosage, effectiveness, and safety of immunosuppressive and biologic therapies. TDM allows physicians to individualize treatment plans, adjust dosages, and switch medications based on real-time monitoring of drug concentrations and response parameters [89]. TDM is particularly useful in IBD, where variability in drug exposure and immunogenicity can influence treatment outcomes.

### 7.5. Nutritional and Lifestyle Interventions

Personalized therapy for complex gastrointestinal disorders includes dietary changes, nutritional supplements, and lifestyle interventions tailored to the patient’s individual needs and preferences. For CeD, for example, personalized nutritional counseling helps patients adopt a GFD, manage nutrient deficiencies, and prevent gluten exposure [54,55,90]. Likewise, in IBD, lifestyle changes such as stress management, exercise, and smoking cessation can complement pharmacological therapy and improve disease progression.

### 7.6. Shared Decision-Making

Shared decision-making involves collaborative discussions between patients, caregivers, and healthcare providers to collaboratively create treatment plans that are consistent with patients’ values, preferences, and care goals, particularly in the pediatric population [91]. Providing information, education, and support to patients enables active participation in treatment decisions, improves adherence to treatment, and improves patient satisfaction and QoL. Overall, personalized therapy for complex gastrointestinal diseases integrates multidimensional approaches including precision medicine, risk stratification and personalized treatment algorithms, therapeutic drug monitoring, nutritional and lifestyle interventions, and shared decision-making. An example of personalized therapy for gastrointestinal disorders in children is the treatment of IBD [91]. Personalized therapy for pediatric IBD involves tailoring treatment strategies to individual patients based on various factors, including disease phenotype, severity, response to treatment, genetics, and patient preferences. Table 2 summarizes the application of personalized therapy in pediatric IBD, one of the best-known paradigms of complex gastrointestinal diseases.

Overall, personalized therapy for complex gastrointestinal diseases involves a multidimensional approach that takes into account disease phenotype, treatment response, genetics, lifestyle factors, and patient preferences. By tailoring treatment strategies to individual patient characteristics and preferences, personalized therapy optimizes outcomes, minimizes side effects, and improves the overall quality of care for patients with complex gastrointestinal diseases.

## 8. The Proteomic Approach in Pediatric Gastroenterology

Proteomics, or the large-scale study of proteins, is critical to advancing our understanding of pediatric gastroenterology by elucidating the molecular mechanisms underlying gastrointestinal diseases, identifying biomarkers for diagnosis and prognosis, and discovering potential targets for personalized therapy. Proteomics can reveal disease causes, discover biomarkers, guide tailored therapy, support drug development, and improve understanding of host–microbiome interactions [92]. Collaboration between physicians and industry partners is critical to translate proteomic discoveries into practical applications that improve outcomes for children with gastrointestinal diseases.

Below are some key applications of proteomics in pediatric gastroenterology.

### 8.1. Disease Mechanisms

Proteomic analyzes enable comprehensive profiling of proteins expressed in gastrointestinal tissues and fluids and shed light on the molecular signaling pathways that are involved in pediatric gastrointestinal diseases such as IBD [93], CeD, EoE, and liver disease. By identifying dysregulated proteins and signaling pathways, proteomics helps elucidate the pathogenesis of these diseases and provides insights into potential therapeutic targets.

### 8.2. Biomarker Discovery

Proteomic techniques such as mass spectrometry and protein microarrays facilitate the discovery of new biomarkers for pediatric gastrointestinal diseases. Biomarkers derived from proteomic analysis of blood, stool, urine, and tissue samples can aid in early diagnosis, disease monitoring, predicting response to treatment, and identifying patients at risk of disease progression or complications [91]. For example, proteomic biomarkers can distinguish between different IBD subtypes (i.e., anti-Saccharomyces cerevisiae antibodies), monitor disease activity (i.e., C-reactive protein, serum amyloid A, fecal calprotectin), or predict the likelihood of response to certain therapies (i.e., antidrug antibodies).

### 8.3. Personalized Medicine

Proteomics contributes to the development of personalized medicine approaches in pediatric gastroenterology by identifying patient-specific molecular signatures and guiding treatment decisions. For example, proteomic profiling of tumor tissues in pediatric gastrointestinal cancers can influence the selection of targeted therapies based on the expression of specific proteins or signaling pathways. Similarly, proteomic analysis of patient samples may help tailor immunosuppressive or biologic therapies for children with IBD or other autoimmune diseases.

### 8.4. Drug Development

Proteomic technologies facilitate drug discovery and development by characterizing drug targets, elucidating drug mechanisms of action, and identifying potential off-target side effects [94]. Proteomic profiling of pediatric patient samples before and after treatment can provide valuable insights into the molecular effects of therapeutic interventions and help optimize drug efficacy and safety in pediatric gastroenterology.

### 8.5. Microbiome Studies

Proteomics complements metaproteomic analysis of the gut microbiome and enables the characterization of microbial proteins and their interactions with host proteins in pediatric gastrointestinal diseases. The integration of proteomic and metaproteomic data provides a comprehensive understanding of host–microbiome interactions and their role in health and disease, offering new opportunities for microbiome-based diagnostics and therapeutics [95]. An example of the use of proteomics in pediatric gastroenterology can be found in a study of IBD, which includes CD and UC [93]. Proteomic techniques have been used to investigate the molecular mechanisms underlying IBD pathogenesis, identify biomarkers for disease diagnosis and monitoring, and uncover potential therapeutic targets (Table 3).

## 9. Conclusions

Treatment options for chronic gastrointestinal diseases, including CeD, EoE, and IBD are evolving with the emergence of novel therapeutic approaches that present challenges to healthcare providers caring for these patients. The available evidence comes largely from adult studies. Many of the therapeutic methods proposed in this manuscript lack sufficient evidence for clinical application with children. In CeD, ongoing research into immunotherapy, zonulin inhibitors, microbiota modulation, nanotechnologies, monoclonal antibodies, and vaccines promise to improve gluten tolerance, reduce intestinal inflammation, and improve patient outcomes. Similarly, investigations into the use of dupilumab and modulation of the thymic stromal lymphopoietin pathway in EoE provide potential opportunities to combat eosinophilic inflammation and restore esophageal homeostasis. Meanwhile, advances in IBD therapeutics, including anti-IL-23 agents, small molecule treatments, and cell-based therapies, offer new strategies to modulate the dysregulated immune response and achieve disease remission. As these innovative treatments progress through clinical trials and enter clinical practice, they may have the potential to revolutionize the treatment of these gastrointestinal diseases and provide patients with treatment options tailored to their specific disease mechanisms and clinical needs. However, further research is needed to clarify the long-term effectiveness, safety, and optimal use of these therapies in pediatric patients and to address challenges such as treatment accessibility, cost-effectiveness, and individual variability in treatment response. Close collaboration between pediatric gastroenterologists, immunologists, researchers, and patient advocacy groups is critical to advancing the field and improving outcomes for children with gastrointestinal diseases.

## Figures and Tables

**Table 1:** Novel biologics for IBD.

	Vedolizumab	Ustekinumab	Risankizumab
Mechanism of action	Targets 47 integrin (inhibiting leukocyte trafficking to the gastrointestinal tract).	Suppresses IL-12 and IL-23, cytokines involved in inflammation.	Targets p19 component of IL-23 cytokine implicated in the pathogenesis.
Indications	Adult patients with moderate to severe CD and UC.	Adult patients with moderate to severe CD and UC.	Clinical trials in CD and UC patients, not yet licensed for IBD therapy.
Clinical trial findings	- High clinical remission rates in pediatric CD (6–17 years) in the VEDOIBD trial. - Positive effects on steroid-free remission and mucosal healing in pediatric CD. - Effective in pediatric UC.	- High clinical remission rates in 12–17 years CD patients (IM-UNITI study). - Limited evidence for pediatric UC (retrospective chart analysis: 9/10 children with UC achieved steroid-free remission).	- Intravenous risankizumab was effective as induction therapy in CD (ADVANCE, MOTIVATE studies). - Subcutaneous risankizumab showed efficacy in sustaining remission in CD (FORTIFY study). - Significant improvement in clinical and endoscopic remission in UC (INSPIRE study).
Pediatric use	Off-label in pediatric IBD.	Off-label in pediatric IBD.	Not yet licensed for pediatric use.

IBD, inflammatory bowel disease; CD, Crohn’s disease; UC, ulcerative colitis.

**Table 2:** Personalized therapy in pediatric inflammatory bowel disease.

Field	Application in IBD
Disease phenotype and severity	Exclusive enteral nutrition for children with mild-to-moderate Crohn’s disease limited to the ileum.
Treatment response and monitoring	Optimization of biological treatment based on therapeutic drug monitoring (through levels and anti-drug antibodies).
Pharmacogenomics	Determination of optimal thiopurine dosage through genetic testing for TPMT and NUDT15 variants.
Nutritional interventions	Nutritional therapies for Crohn’s disease (i.e., exclusive enteral nutrition, Crohn’s disease exclusion diet).
Lifestyle	Smoke as risk factor for disease relapse.
Shared decision-making	Improving long-term treatment adherence by involving pediatric patients and their families in treatment decisions.

IBD, inflammatory bowel disease; TPMT, thiopurine S-methyltransferase, NUDT15, nudix hydrolase 15.

**Table 3:** Application of proteomics in pediatric IBD research.

Field	Strategy Applied	Goal
Disease mechanisms	Proteomic analyses of intestinal tissues and immune cells	Insights into the dysregulated signaling pathways and immune responses that drive disease pathogenesis.
	Profiling the expression levels of proteins involved in inflammation, immune regulation, barrier function, and tissue remodeling	To elucidate the molecular mechanisms underlying the development and progression of IBD.
Biomarker discovery	Proteomic analyses of serum, fecal, or mucosal samples	Discovery of differential expression of proteins associated with inflammation (i.e., cytokines, acute phase reactants), intestinal barrier integrity (i.e., tight junction proteins), and microbe-host interactions (i.e., antimicrobial peptides).
Personalized medicine	Proteomic profiling of mucosal samples	Identification of subgroups of patients with different molecular phenotypes or treatment responses.
Drug development	Identification of dysregulated proteins and signaling pathways associated with disease pathogenesis	Prioritize potential targets for drug interventions and optimize therapeutic strategies.
	Monitoring changes in protein expression profiles in response to treatment	Evaluation of drug efficacy and safety

IBD, inflammatory bowel disease.

## Data Availability

Not applicable.

## Abbreviations

The following abbreviations are used in this manuscript:

AIH	Autoimmune hepatitis
CeD	Celiac disease
EoE	Eosinophilic esophagitis
IBD	Inflammatory bowel disease
ESPGHAN	European Society of Pediatric Gastroenterology, Hepatology and Nutrition
GFD	Gluten-free diet
PEP	Propylendopeptidase
EP	Endoprotease
TG	Transglutaminase
JAK	Janus kinase
QoL	Quality of life
PEG	Polyethylene glycol
OIT	Oral immunotherapy
SLIT	Sublingual immunotherapy
GSRS	Gastrointestinal Symptom Rating Scale
FMT	Fecal microbiota transplantation
NAFLD	Non-alcoholic fatty liver disease
IL	Interleukin
TSLP	Thymic stromal lymphopoietin
CD	Crohn’s disease
UC	Ulcerative colitis
TDM	Therapeutic drug monitoring
VEO-IBD	Very early onset inflammatory bowel disease
HLA	Human leukocyte antigen
TPMT	Thiopurine S-methyltransferase
NUDT15	Nudix hydrolase 15
